# Study on the mechanical properties of lithium slag recycled fine aggregate concrete

**DOI:** 10.1371/journal.pone.0326925

**Published:** 2025-06-30

**Authors:** Jun Wan, Caisen Wang, Jiongfeng Liang, Yunchen Wang

**Affiliations:** 1 College of Architecture Engineering, Jiangxi Science and Technology Normal University, Nanchang, China; 2 College of Architecture and Civil Engineering, Beijing University of Technology, Beijing, China; 3 School of Civil & Architecture Engineering, East China University of Technology, Nanchang, China; SASTRA Deemed University, INDIA

## Abstract

To promote the sustainable utilization of industrial solid wastes in concrete applications, this study systematically investigates the combined use of lithium slag (LS) as a cement replacement and recycled fine aggregates (RFA) as a substitute for river sand (RS). Through experimental analysis with a fixed water-cement ratio (0.46), we evaluated the effects of varying LS content (0–40%) and RFA replacement rates (0–30%) on concrete performance. The results indicate that the optimal LS incorporation (20%) enhances compressive strength, splitting tensile strength, and flexural strength by 12.7%, 11.9%, and 9.16%, respectively, while maintaining adequate workability. In contrast, RFA addition caused a linear reduction in mechanical properties, with 30% RFA leading to a 19.07% decrease in compressive strength. However, the addition of LS effectively mitigated the performance losses induced by RFA, providing a compensatory effect. The conversion formulas established between cubic compressive strength and other mechanical parameters demonstrated high correlation coefficients, offering practical guidelines for lithium slag-recycled fine aggregate concrete (LSRFAC) applications. This dual-waste utilization strategy presents an environmentally responsible solution for construction material innovation, addressing both the recycling of industrial byproducts and the conservation of natural resources. Overall, this study provides a sustainable approach to concrete production by reducing environmental burdens and supporting the circular use of industrial and construction waste in structural engineering.

## 1. Introduction

As the global economy continues to expand, urban construction is accelerating, leading to increasing demand for building materials. Due to its wide availability and favorable mechanical properties, concrete has become the preferred choice for engineers [1,2]. However, the excessive extraction of raw materials such as limestone, clay, river sand (RS), and gravel has caused serious ecological damage and environmental imbalance. In response, many countries are promoting the use of “green” concrete by incorporating industrial and construction waste, such as electric arc furnace dust [3], wood ash [4], silica fume [5], fly ash [6], copper tailings [7], waste rubber [8], and recycled aggregates derived from demolition waste [9–12].

In China, lithium slag (LS) is a byproduct of lithium carbonate production via the sulfuric acid process, generated during the calcination of corundum ore at 1200 °C. For each ton of lithium carbonate produced, 8–10 tons of LS are generated [13]. LS is primarily used in the production of rechargeable batteries and lithium hydroxide (LiOH) for the ceramics industry [14,15]. With an annual output exceeding 240,000 tons, production is regionally concentrated in provinces such as Jiangxi and Xinjiang [16]. Most LS is still landfilled, which poses long-term environmental risks [17].

LS mainly consists of SiO₂ and Al₂O₃, giving it cementitious potential [18,19]. He et al. [13] found that using LS as a cement substitute improves concrete microstructure and densifies the interfacial transition zone. Yang et al. [20] reported that replacing 20% of cement with LS in ultra-high-performance concrete (UHPC) resulted in a compressive strength of 134.48 MPa after 28 days. Rahman et al. [18] similarly observed enhanced mechanical strength at 40% LS substitution.

However, while LS and RFA have each been studied independently as alternative materials in concrete, their combined application remains underexplored. In particular, there is limited research on how varying substitution rates of LS and RFA jointly affect both workability and mechanical performance under a consistent mix design. This study addresses this gap by systematically investigating their synergistic effects in concrete, thereby supporting the development of more sustainable and resource-efficient construction materials. At the same time, construction waste has become a major source of pollution, accounting for 30–40% of global solid waste [20]. While some developed countries achieve nearly 100% utilization, China’s recycling rate remains at 20–30% [21]. Though RFA derived from waste concrete offers environmental benefits, its lower quality limits engineering applications [22–24]. Improvement methods such as mechanical grinding [25], acid treatment [26], and thermal processing [27] have shown promise. Bayraktar et al. [28] reported that incorporating steel fibers enhances the tensile and flexural strength of RFA concrete. Chen et al. [29] demonstrated that using 20% RFA with particle sizes of 1.18–2.36 mm in UHPC improved compressive strength by 7.1%. Nili et al. [30] found that RFA substitution below 30% does not significantly compromise mechanical properties, while Cheng et al. [31] highlighted the potential of graphene oxide to mitigate strength loss. The aim of this study was to investigate the use of LS and RFA in concrete and to assess their impact on the mechanical properties of concrete. The study fills the gap in the joint use of LS and RFA and provides theoretical support for the development of sustainable building materials.

Although the application of LS and RFA as replacement materials in concrete has been investigated separately, the combined effects of their combined use on the workability and mechanical properties of concrete have not been fully explored, especially the synergistic effects at different replacement rates. Thus, this study addresses the research gap by investigating the combined use of lithium slag (LS) and recycled fine aggregate (RFA) in concrete. Under a fixed water–cement ratio of 0.46, LS is used to partially replace cement (at 0%, 10%, 20%, and 40%), while RFA replaces river sand at 0%, 10%, 20%, and 30%. The workability and mechanical properties of the resulting concrete mixtures are evaluated.

## 2. Materials and methods

### 2.1. Materials

#### 2.1.1. Cement and LS.

**Fig 1:**
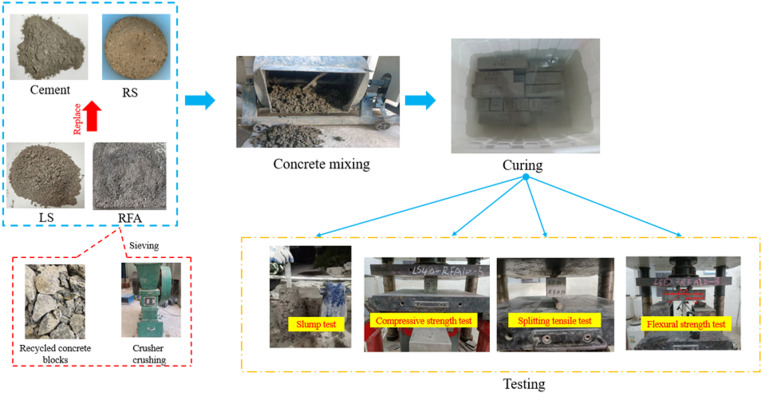
Experiment process.

Lithium slag is a byproduct of lithium carbonate production via the sulfuric acid process, primarily generated in provinces such as Jiangxi and Xinjiang. For each ton of lithium carbonate, 8–10 tons of LS are produced. The LS used in this study had an average particle size of 13.21 μm, and its appearance is shown in Fig 1. The chemical composition of LS, presented in Table 1, indicates that it is mainly composed of SiO₂ (45.90%), Al₂O₃ (19.3%), and CaO (9.70%), reflecting its potential pozzolanic activity due to high silicate and aluminate contents.

**Table 1:** List of chemical composition for cement and LS (wt%).

Material	SiO_2_	CaO	Al_2_O_3_	Fe_2_O_3_	MgO	TiO_2_	SO_3_	Other
cement	61.62	21.01	4.55	3.43	1.27	0.07	2.38	5.67
LS	45.90	9.70	19.3	1.23	1.10	2.20	5.97	14.6

**Fig 2:**
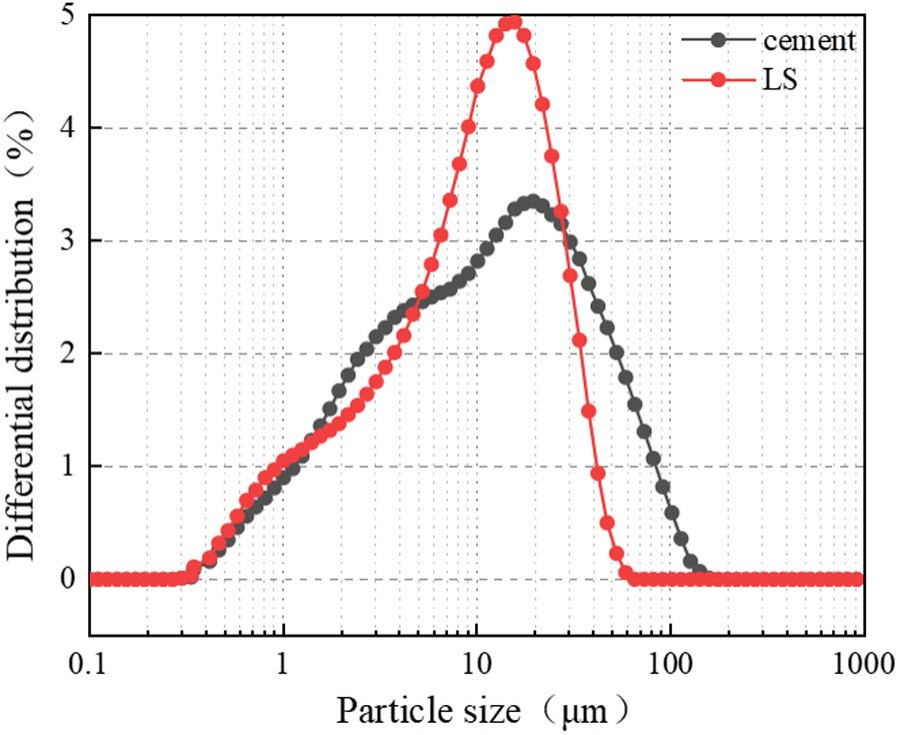
Cement and LS particle size distribution.

LS was selected in this study to partially replace cement because of its cementitious properties. It can react with Ca(OH)₂ from cement hydration to produce calcium silicate hydrate (C-S-H) and calcium aluminate hydrate (C-A-H), enhancing concrete microstructure and mechanical performance. The particle size distribution of cement and LS is shown in Fig 2.

#### 2.1.2. Aggregates.

**Fig 3:**
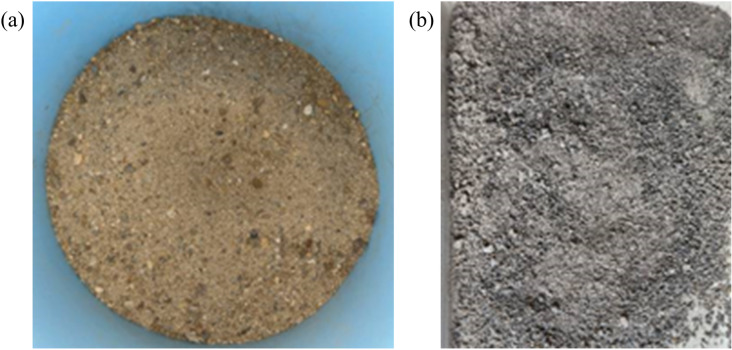
Natural appearance of RS and RFA. **(a)** RS, **(b)** RFA.

**Fig 4:**
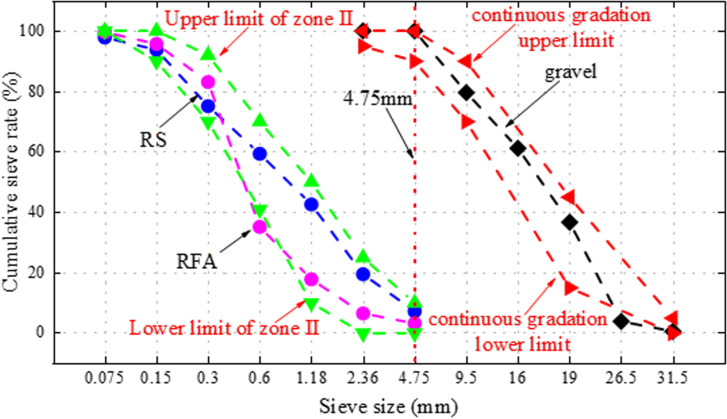
Gradation curve of RS, RFA and gravel.

RS: The RS has a modulus of fineness of 2.74, which is within the range of medium sands with excellent particle size, a bulk density of 1479 kg/m^3^, an apparent density of 2497 kg/m^3^, and a water absorption of 2.31%, the natural appearance of RS is depicted in Fig 3(a), and the particle size sieving curve is depicted in Fig 4.

RFA: The RFA from the waste pavement concrete of East China University of Technology was used in this study, aiming to realize the resourceful use of construction waste and alleviate the over-exploitation of the natural river sand resources. The use of RFA helps to reduce the landfill of construction waste and promotes the closed-loop use of resources, which is in line with the goals of green building and sustainable development. The roadway was originally a plain C30 grade concrete with a service life of approximately 15 years. After demolition, the concrete blocks were hand-selected with no visible contamination or carbonation, and then crushed and screened in the laboratory to obtain recycled fine aggregates with a particle size of less than 4.75 mm and a fineness modulus of 2.18. No other raw materials were added during the whole process to ensure the reproducibility of the research data and the representativeness of the materials. An apparent density of 2361 kg/m^3^and a bulk density of 1346kg/m^3^, however, the water absorption rate is 9.78%, which is more than to 4 times, the water absorption rate of RS. According to Japan’s standards for recycled aggregates, RFA with a water absorption rate of more than 13% are not recommended in concrete production [32]. In order to avoid excessive water absorption of the RFA that could affect testing results, the day before the test, place the RFA in water for 24 hours.

The natural appearance of RFA has been illustrated in Fig 3(b) and the gradation curve of RS, RFA and gravel is illustrated in Fig 4.

Coarse aggregates: An apparent density of 2942 kg/m^3^, a bulk density of 1679 kg/m^3^ and a water absorption of 1.38%, the crushing index value was 7.9%.

Water: The water used for testing was laboratory water, which has been tested by relevant institutions and has a pH value of 7.24, which meets the requirements of JGJ63–2006 specification.

### 2.2. Mix design and test methods

Considering the impact of two factors: the LS content (0, 10%, 20%, 40%) and the replacement rate of RFA (0, 10%, 20%, 30%), a series of 16 sets of test blocks with different mixing ratios were designed, and 96 non-standard cubic test blocks of 100 mm × 100 mm × 100 mm were produced, used to test the compressive strength and splitting tensile strength of concrete cubes 28 days later; furthermore 48 specimens of 100 mm × 100 mm × 400 mm non-standard prism test block to obtain the flexural strength of concrete after 28d. There are certain conversion coefficients between the cubic compressive strength, splitting tensile strength and flexural strength of the calculated standard test block and the non-standard test block, which are 0.95, 0.85 and 0.85, respectively. The test design strength grade was 30MPa, and the concrete design slump value was 75–90 mm. Table 2 lists the specific mix pro port.

**Table 2:** Composition of concrete (kg/m^3^).

LS (%)	RFA (%)	Cement	LS	RS	RFA	Water/ Gravel
0	0	446.0	0.0	525.0	0.0	205/ 1224
	10	446.0	0.0	472.5	52.5	205/ 1224
	20	446.0	0.0	420.0	105.0	205/ 1224
	30	446.0	0.0	367.5	157.5	205/ 1224
10	0	401.4	44.6	525.0	0.0	205/ 1224
	10	401.4	44.6	472.5	52.5	205/ 1224
	20	401.4	44.6	420.0	105.0	205/ 1224
	30	401.4	44.6	367.5	157.5	205/ 1224
20	0	356.8	89.2	525.0	0.0	205/ 1224
	10	356.8	89.2	472.5	52.5	205/ 1224
	20	356.8	89.2	420.0	105.0	205/ 1224
	30	356.8	89.2	367.5	157.5	205/ 1224
40	0	267.6	178.4	525.0	0.0	205/ 1224
	10	267.6	178.4	472.5	52.5	205/ 1224
	20	267.6	178.4	420.0	105.0	205/ 1224
	30	267.6	178.4	367.5	157.5	205/ 1224

**Fig 5:**
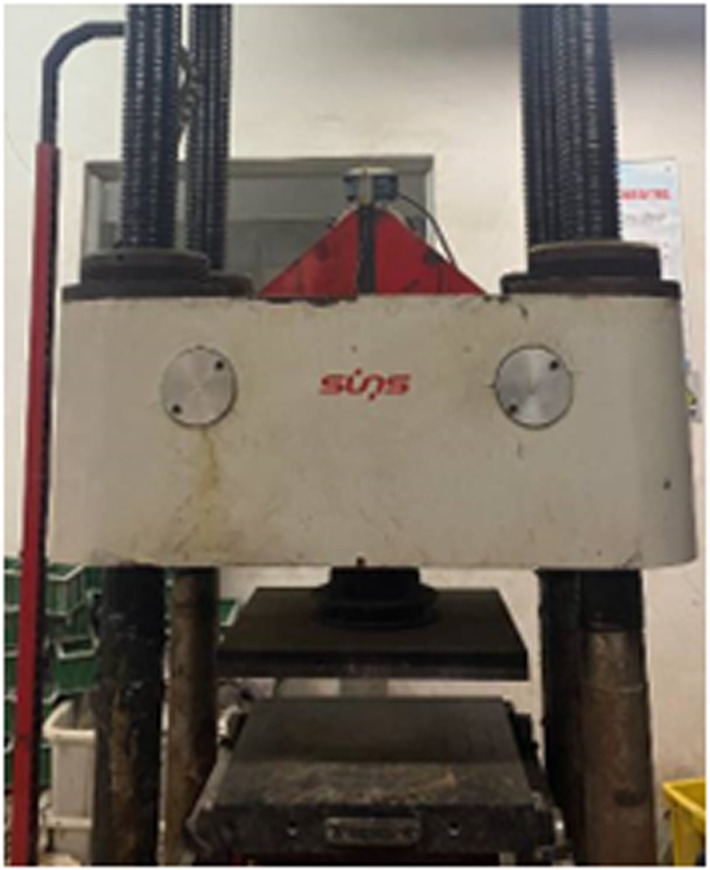
YAW-3000 type test equipment.

A pressure tester with a maximum pressure range of 3000kN was used for the tests, as shown in Fig 5. The test was conducted according to the requirements of GB/T 50080−2002 [33] Standard Test Methods for Ordinary Concrete Mixes and GB/T 50081−2019 [34] Standard Test Methods for Physical and Mechanical Properties of Concrete. In order to reduce the test error, all samples were maintained in a constant temperature and humidity environment, and the RFA was pre-soaked in water for 24 hours before the test to prevent water absorption from affecting the slump. At least 3 specimens were used in each test group and the average value was taken to ensure the repeatability and representativeness of the test results.

## 3. Results of the tests and discussion

Although this study provides valuable data on the effectiveness of LS and RFA in concrete, it is recognized that this study was carried out on a laboratory scale and therefore the results may be affected by different factors in practical engineering applications. In addition, RFA is inherently more variable, and this variability may affect the performance of concrete in practical applications. These potential variables and practical operating conditions must be considered when applying the results of these studies to larger-scale construction projects.

### 3.1. Slump

**Fig 6:**
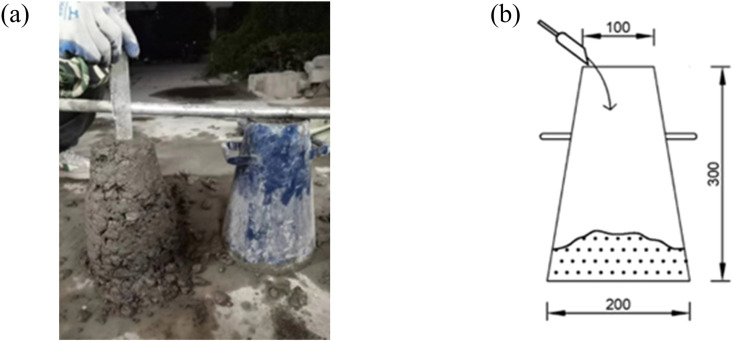
Slump test.

The slump test diagram for LSRFAC is shown in Fig 6. First of all, the inner wall of the slump cylinder used in the test is coated with edible oil to reduce the friction of the internal cylinder wall and affect the test results. Then fill the slump cylinder with concrete in equal parts 3 times, after the three sub-assembly and the insertion is completed, the mouth of the slump cylinder is smoothed with a spatula, and then the excess concrete around the slump cylinder is removed, and then the concrete slump cylinder is lifted vertically upwards immediately, when the concrete no longer collapses downward, the iron rod of the configuration is placed above the slump cylinder, and then the concrete drop value is measured with a ruler, and the slump value reading is recorded at this time.

When performing a slump test, It can be found that when LS was added to concrete within 20%, during mixing of the mixture, the insertion resistance is comparable to the insertion resistance of the reference concrete and the insertion process of the slump, and there was no significant change; with further rise in LS content, it was obviously felt that the insertion resistance was slightly increased at this time, and the fluidity of the mixture was also worse. However, when RFA was added to replace RS for mixing, it was found that the resistance was higher than the benchmark concrete group during the insertion and tamping process, and the fluidity of the mixture became worse. With the rise of the replacement rate of RFA, the mixing resistance was also gradually increased. Especially when the replacement rate of RFA reached 30%, it was obviously more difficult to insert and tamp. However, when LS and RFA were added at the same time, LS with an appropriate amount could improve the fluidity of LSRFAC mixture.

**Fig 7:**
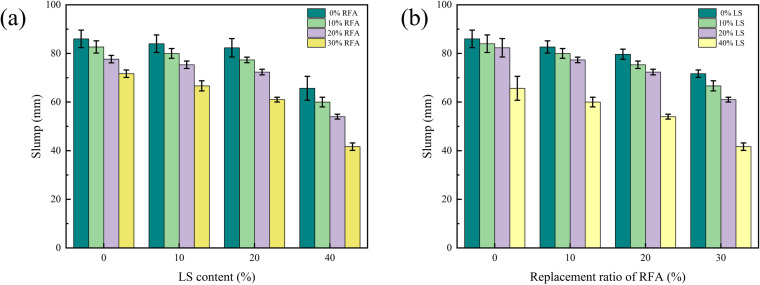
Effect of LS content and replacement rate of RFA on the slump.

It is clear from Fig 7 that at 0% substitution rate of RFA, the slump of concrete mixed with 10%, 20% LS decreased slightly with that of concrete 0% LS; it was basically no difference. However, the slump value of concrete mixed with 40% LS dropped sharply to 68 mm, which was 21.83% below the concrete of the reference group. The reason may be that when LS was used to replace cement in proportion, the cement is slightly smaller than that of the LS. At this time, more cement slurry was used to wrap around LS particles, which showed that the fluidity of LS concrete was reduced. At the same time, LS had a ball effect, so it could act as a ball to increase the fluidity of LS concrete. The slight increase in workability at lower LS contents is attributed to the so-called ‘ball effect,’ where fine, smooth LS particles act similarly to micro ball bearings, reducing interparticle friction and enhancing mixture flowability. The fluidity of LS concrete was determined by the above two effects [35,36]. When the LS content was less than 20%, the slump value was almost the same as that of concrete 0% LS. At this time, LS was more reflected as a ball effect to maintain the concrete slump. However, at 40% LS, the cement content was reduced, which was not enough to provide the needed cement paste to maintain the LS coating, which was directly reflected in the reduction of concrete slump value.

The slump of RFA concrete shows a decreasing trend as the RFA replacement rate increases and the slope of the decreasing section is increasing, which is shown in Fig 7. Compared with the benchmark concrete with 0% LS content, the slump value of concrete with 10%, 20% and 30% replacement rate of RFA decreased by 4.59%, 10.34% and 17.24% respectively. The reason may be that when the RFA replaces the RS in proportion, the RFA has many edges and corners, rough surface, and a significant amount of micro-cracks would be caused inside the RFA during the crushing process, which leaded to a large water absorption rate of the RFA [37,38], which required more water to make up for the defects of the RFA. At this time, the concrete slump value directly decreased.

When the LS content was 0%, the addition of RFA led to a decrease in the concrete’s slump. However, the resulting slump still met the design requirements. When the LS content was less than 20%, the slump value of RFA concrete with 30% substitution rate dropped below 70 mm, which did not met the design requirements; when the LS content was 40%, the slump of concrete with or without RFA was far from the design requirements. This may be LS has the dual characteristics of reducing the fluidity of the mixture and ball effect, while the RFA has natural defects such as many particle edges and corners, rough surface, etc. When the two acted together, the right amount of LS can compensate for reduced mobility due to RFA defects. However, when the LS content exceeded 20%, LS replaced too much cement so that it could not produce enough slurry to maintain the mixing flow. However, the RFA has large water absorption after crushing, so the concrete slump cannot meet the requirements of design specifications.

### 3.2. Compressive strength

During the compressive and flexural tests, the failure of the specimens was sudden and accompanied by crack initiation and rapid propagation perpendicular to the loading axis, indicating a typical mode brittle fracture. While this study did not directly measure fracture energy using standardized fracture mechanics tests, the observed conical failure in compressive specimens and mid-span rupture in flexural beams suggest a low energy dissipation capacity typical of brittle materials. Future studies should include fracture energy testing to quantify the failure toughness of LSRFAC.

**Fig 8:**
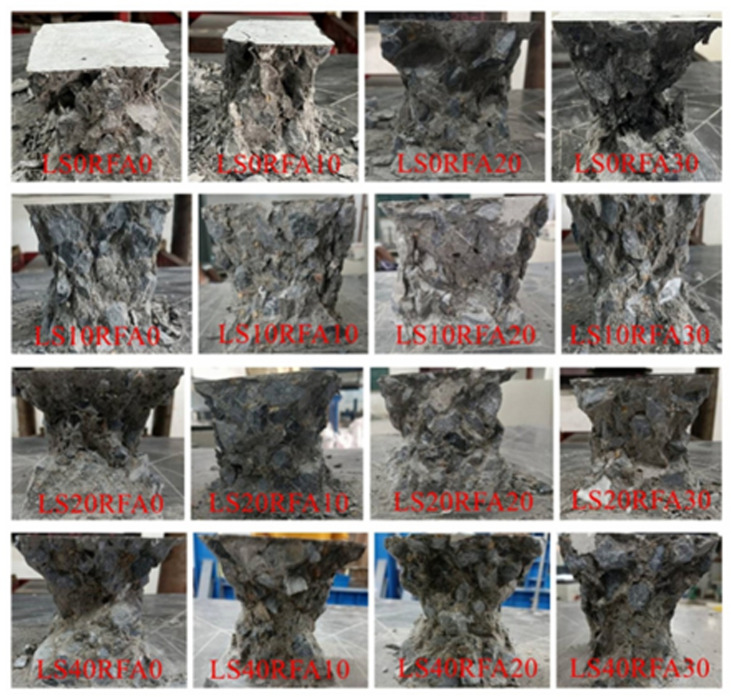
Failure diagram of a concrete cube compressive test block.

**Fig 9:**
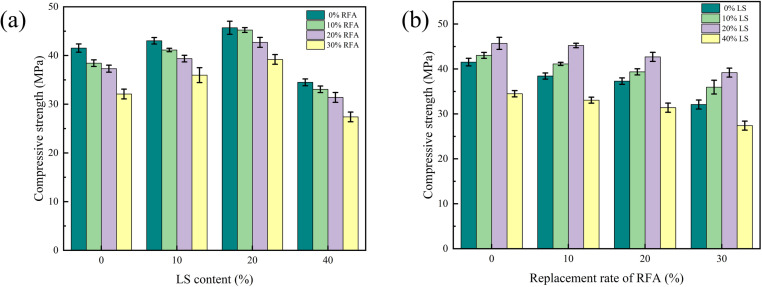
Effect of LS content and replacement rate of RFA on the compressive strength.

The failure diagram of all concrete cube test blocks is shown in Fig 8. It can be found from when the test specimens started to pressurize, its surface had no obvious change. When the load further increased, the test specimens made an insignificant “squeak” sound. Besides, the concrete specimen surface began to appear irregular small cracks. The number of cracks increases with increasing loads. The cracks in the corners of the walls are starting to connect with the cracks on the other sides of the wall, furthermore the surface concrete had an unimportant fall off, until the final failure accompanied by brittle sound. Finally, the specimen showed a conical failure shape. The failure forms of LS and benchmark concrete were basically similar. Cracks on concrete surfaces increased as the RFA concrete replacement rate increased and showed an irregular shape, and the development of cracks almost happened at the same time. When the load capacity of the specimen reached the limit value, the surface of the concrete would peel off, accompanied by gravel falling. By observing the internal damage of LSRFAC, it can be found that the crushed stone inside the concrete was rarely crushed, and most of the damage of the test specimens was caused by the separation between the crushed stone and the cement due to insufficient cohesion. The relationship between LS content, RFA replacement rate and concrete compressive strength depicted in Fig 9. The results of the calculations are shown in Table 3.

**Table 3:** Specimen test results.

LS content%	Replacement rate of RFA%	Slump (mm)	Compressive strength (MPa)	Splitting tensile strength (MPa)	Flexural strength (MPa)
0	0	87	40.9	2.34	4.17
0	10	83	39.2	2.24	4.00
0	20	78	36.5	2.11	3.86
0	30	72	33.1	1.98	3.64
10	0	85	43.6	2.47	4.37
10	10	80	41.5	2.39	4.23
10	20	74	39.9	2.26	4.05
10	30	65	36.3	2.07	3.77
20	0	84	46.1	2.62	4.56
20	10	78	45.0	2.54	4.45
20	20	71	42.9	2.40	4.32
20	30	61	39.2	2.21	4.10
40	0	68	33.8	1.78	3.75
40	10	60	32.9	1.65	3.62
40	20	53	31.2	1.52	3.46
40	30	40	26.4	1.31	3.15

As displayed in Fig 9, the compressive strength of concrete increases gradually with the increase of LS content and reaches the peak at 20% LS content, then decreases gradually with the increase of LS content. The compressive strength of 40% LS concrete is 17.36% lower than that of 0% LS at 0% replacement of RFA. The reason may be that LS has pozzolanic effect [39], and its components contain more SiO_2_ and Al_2_O_3_, which can react with the hydration product Ca(OH)_2_ in cement produces more hydration to fill the pores of the concrete, thus reducing the porosity of the concrete [36]. However, when the LS content is further increased, the secondary hydration products generated during this time cannot make up for the hydration products generated by LS replacing cement in an equal amount, which leads to the rise of pores in concrete and reduces the compressive strength of concrete [40]. This trend is consistent with Yang et al. (2024), who reported similar improvements in compressive strength up to 20% LS in UHPC [20]. The decline at higher LS levels is also in agreement with Rahman et al.,who attributed the loss to insufficient cementitious reaction products [41].

From Fig 9, it is observed that the compressive strength of RFA concrete tended to decline as the RFA replacement rate increased. When the content of LS was 0%, the replacement rate of RFA was 30%, its compressive strength decreases by 19.07%. The main reasons may be that the roughness of the RFA surface, contains many micro cracks, and has poor solidity. The strength of the RFA itself is not as strong as that of RS, and the gap between the coarse aggregates cannot be effectively filled [42,43]. Consequently, the compactness of the concrete decreases, the porosity increases, and stress concentration is easy to occur when the concrete is compressed, which is the main cause of the above phenomenon. These results align with Nili et al. and Cheng et al., who found that increased RFA content reduces compressive strength due to the high porosity and poor bonding characteristics of recycled aggregates [30,31].

According to Fig 9, when LS and RFA were added to the concrete together, only the specimen LS40RFA30 did not meet the design strength 30MPa requirements, and the rest of the specimens meet the design strength requirements. According to the compression strength results of the reference concrete, the specimens LS10RFA0, LS10RFA10, LS20RFA0, LS20RFA10 and LS20RFA20 were higher than the compression test results of the reference group. This showed that an appropriate amount of LS could make up for the defect that RFA was easy to crush, but the reduction of compressive strength would increased sharply when excessive LS was mixed with a RFA. This was because as the two materials were added together to the concrete, the right amount of LS can create a hydration reaction that produces material to fill some of the small cracks in the RFA. As the replacement rate of RFA increases, the hydration products produced by LS were not enough to fill the small cracks, so the pressure resistance showed a downward trend. When the LS content was more than 20%, the cement content decreases with increasing LS. At this time, the compressive strength had been reduced because it was not enough to make up for the hydration products produced by the cement. If the replacement rate of RFA continued to increase, the compressive strength would showed a greater decline.

### 3.3. Splitting tensile strength

#### 3.3.1. Results and discussion of splitting tensile strength.

**Fig 10:**
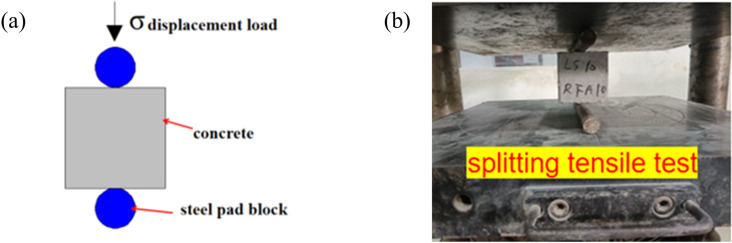
Loading diagram of splitting tensile test.

**Fig 11:**
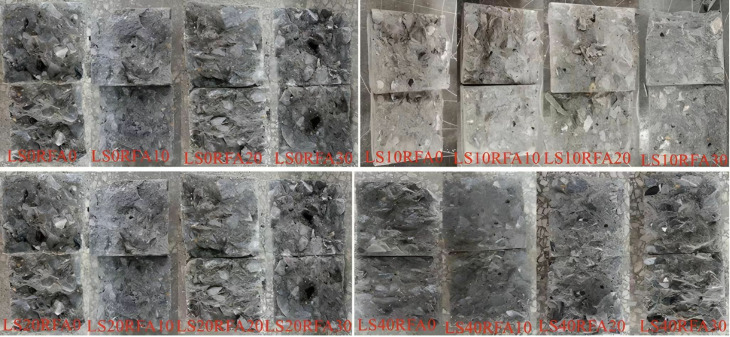
Failure diagram of concrete splitting tensile test block.

Fig 10 shows the loading schematic for the splitting tensile strength test of the LSRFAC, and the field test is shown in Fig 10. Observe the splitting tensile test process of concrete specimens, and there was no obvious cracks on specimen surface when loading starts. As the increase of load, irregular micro-cracks started to emerge on the surface of the specimen, which became more and more obvious with the increase of the continuous compression load. The cracks were mainly distributed in the middle of the test specimens. When the pressure reached the limit, the concrete specimen was accompanied by a brittle sound, and divide the specimen into two halves along the upper and lower curved pads. Through observation, it found that the failure form of LS and RFA concrete was very similar to the form and process of reference concrete. The only difference was that more powder and stone chips would appear during the splitting process of concrete with RFA. The failure diagram of the splitting tensile test of LSRFAC test block is shown in Fig 11.

**Fig 12:**
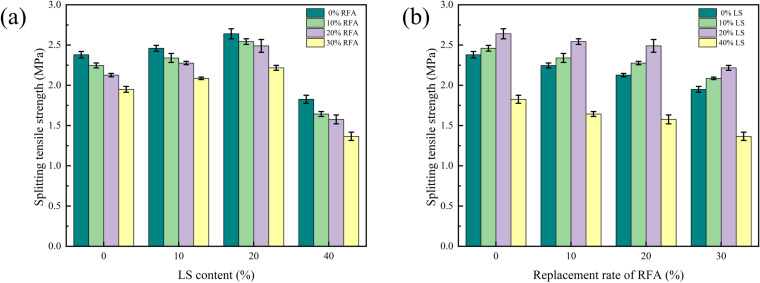
Effect of LS content and replacement rate of RFA on the splitting tensile strength.

From Fig 12, it can be seen with the addition of LS content, the splitting tensile strength of concrete gradually increasing and reaching the peak at 20% LS content, and then continued to increase the LS content, the tensile strength also showed a downward trend. At 0% RFA, the splitting tensile strength of concrete with 40% LS was 23.9% lower than that without LS. This is because of the Pazolam reaction between LS and Ca(OH)_2_ liberated by cement hydration, which generates stable C-S-H gel and hydrated calcium aluminate, which consumes Ca(OH)_2_ at the same time, it also promotes the hydration reaction of cement, which is conducive to the bonding of the concrete interface [12,35,40,41,44]. At the same time, LS also contains more sulfate ions, which is easy to combine with hydrated aluminate calcium to form dense substances, which are filled in the concrete pores to make them more dense. Therefore, the splitting tensile strength of concrete has been greatly enhanced. When the LS content was 40%, the hydration products generated by cement were seriously insufficient, leading to the increase of porosity, to reduce the splitting tensile strength of concrete.

As can be seen in Fig 12, the splitting tensile strength of RFA concrete tended to decrease as the RFA replacement rate increased, and the greater the amount of recycled fine aggregate used, the more noticeable the downward trend was. When the content of LS was 0%, the splitting tensile strength of RFA concrete with 30% substitution rate decreased by 15.38% compared with that without LS. The reason analysis is that the RFA and RS cannot form a good particle grading, and unable to efficiently fill the voids between coarse aggregates, which leads to the increase of the porosity of the concrete [21,38,44]. Furthermore, the hydration of the new and old cement mortar of the RFA is not sufficient, which has adverse effects on the weak area at the interface, and the cohesiveness between the aggregate and the cement becomes worse. Therefore, when the concrete specimen was under load, The micro cracks and weak interfaces in the test specimen first produce failure. Therefore, when the amount of RFA increased, the splitting tensile strength of concrete decreased continuously, these findings are consistent with studies by Zhou et al. and Tam et al., who also observed weakening in tensile properties with high RFA substitution [22,23].

It found that the addition of LS with the content of less than 20% could compensate for the shortcomings caused by the use of RFA instead of RS, while the splitting tensile strength of LS with the content of 40% had a downward trend, and the continued addition of RFA would made its splitting tensile strength show a greater decline. The mechanism analysis is that when lithium slag replaces cement and acts on concrete, on the one hand, some chemical components in LS can react with the hydration products in cement to form C-A-H and C-S-H, so as to achieve the purpose of refining the concrete pore structure. On the other hand, the C-S-H with a low Ca/Si ratio generated by hydration is more stable than the C-S-H with a high Ca/Si ratio when the Ca/Si ratio of the reactants is low [45]. Meantime, it may be that unreacted LS can form a good particle gradation with a RS and RFA. Therefore, the right amount of LS can overcome the shortcomings caused by the replacement of the RFA by the RS, making the splitting tensile strength slightly increased. When LS is further increased, because it cannot provide enough hydration products to fill the concrete pores, and the RFA has some defects, when the two materials work together, the splitting tensile strength of concrete is reduced significantly.

LS was able to react with calcium hydroxide in the cement through volcanic ash reaction to produce more hydrated calcium silicate (C-S-H) gels, thus increasing the compressive and tensile strength of the concrete. However, when the replacement ratio of lithium slag is more than 20%, the generation of cement hydration products is insufficient due to the lack of sufficient calcium source to participate in the reaction, which leads to an increase in the internal porosity of the concrete and thus reduces the strength of the concrete. Therefore, a reasonable amount of LS addition is essential to improve the concrete properties.

#### 3.3.2. Splitting tensile is related to the compressive strength of the cube.

**Fig 13:**
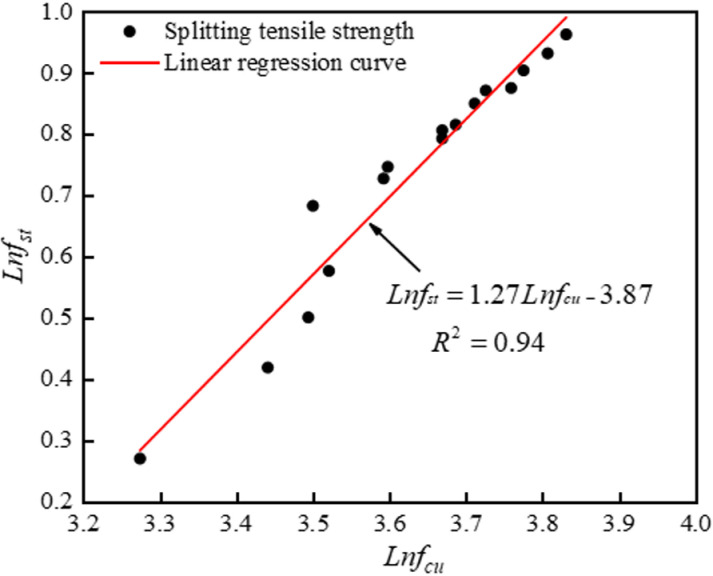
Splitting tensile strength versus cubic compressive strength.


According to the research results of many scholars at home and abroad, the splitting tensile strength value of concrete is generally 1/20–1/10 of the cube compressive strength value. In the relevant code [46], the relationship between the cubic compressive strength (*f*_*cu*_) and the splitting tensile strength (*f*_*st*_) is in accordance with: $\begin{array}{c} f_{st}=0.19f_{cu^{3/4}} \end{array}$ , Song et al. [47] concluded that the relationship between the *f*_*cu*_ and the *f*_*st*_ of the concrete cube of lithium slag recycled coarse aggregate was as follows: $\begin{array}{c} f_{st}=0.168f_{cu^{0.80153}} \end{array}$. In this paper, the *f*_*st*_ and the *f*_*cu*_ of LSRFAC are taken as logarithms respectively, and the logarithm of *f*_*st*_ and *f*_*cu*_ is performed with the help of a linear function relationship model, as shown in Fig 13. Eq. (1) is obtained by slightly deforming the linear function relation obtained by linear regression:

$$\begin{array}{c} f_{st}=0.02f_{cu^{1.27}} \end{array}$$ (1)

**Fig 14:**
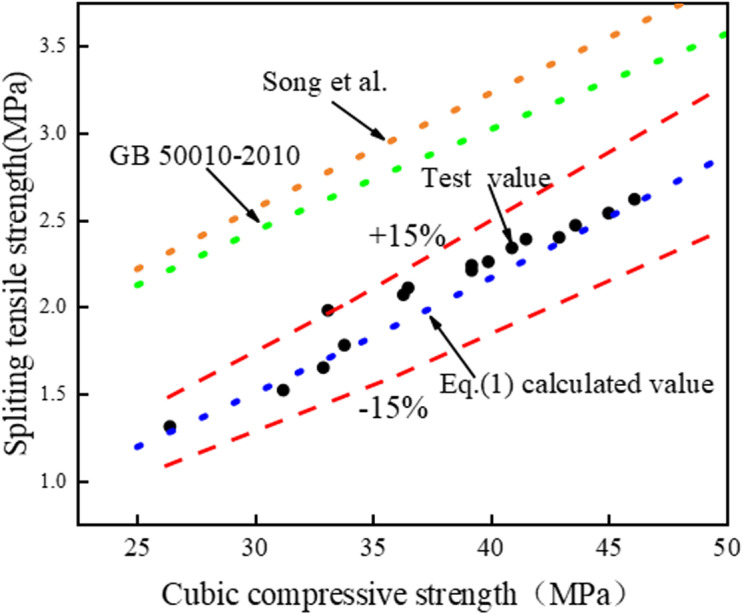
Comparison of splitting tensile strength values.

This can be observed in Fig 13, the experimental value of the splitting tensile strength is in good agreement with the linear regression curve, R^2^ = 0.95, the error value of the two is basically within ±15%. In order to facilitate the reader’s distinction, this paper compares the test value of splitting tensile strength of LSRFAC, the calculated value of Eq. (1), the formula proposed by Song et al., and the standard value of GB 50010−2010, as indicated in Fig 14, where the calculated value of Eq. (1) differs considerably from the standard value of Song et al., and GB 50010−2010, which also indicates that the empirical formula proposed by the previous author may not be applicable to LS and RFA concrete materials.

### 3.4. Flexural strength

#### 3.4.1. Flexural strength results and discussions.

**Fig 15:**
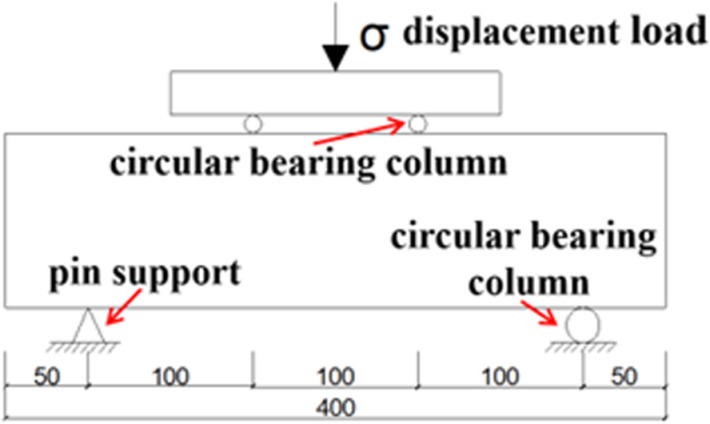
Schematic diagram of bending test block.

**Fig 16:**
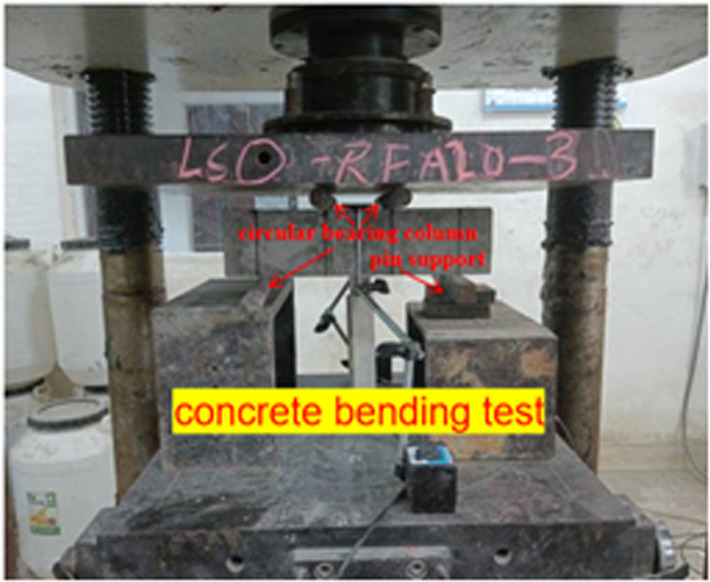
Concrete bending test.

**Fig 17:**
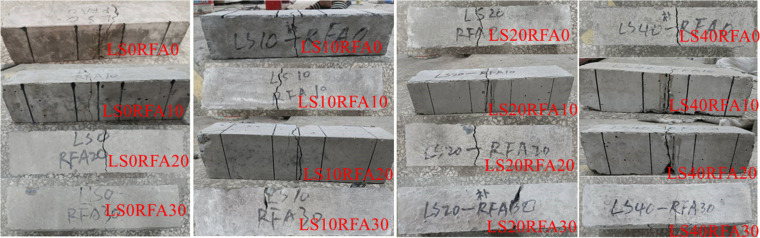
Failure diagram of concrete flexural test block.

The loading schematic diagram of LSRFAC bending test is shown in Fig 15, and the field test is shown in Fig 16. Through observation we can see that the failure process of the concrete test specimen bending test that when the testing machine started to load, there was no change on the surface of the concrete test specimen. With increasing load, microcracks appeared on the surface of the test specimen. With the development of micro cracks, accompanied by a clear sound, the bending test specimen suddenly braked from the middle without any sign, and some debris would be produced during the failure. Through observation, it found that the failure mode and process of LS and RFA concrete were very similar to those of the benchmark concrete, both of which were from the middle without signs of fracture and show obvious brittle failure. The difference was that when the amount of RFA increased, the debris dropped more when the test specimen was broken. The failure diagram of LSRFAC flexural test block is shown in Fig 17.

**Fig 18:**
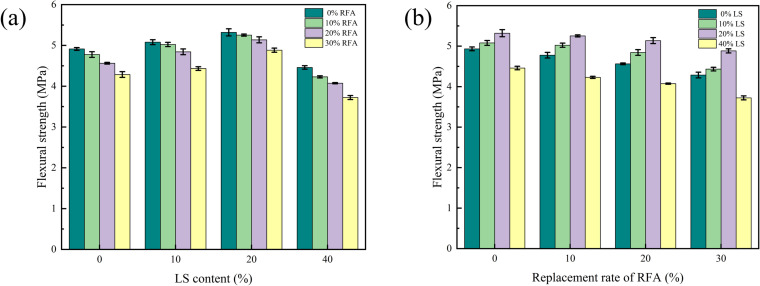
Effect of LS content and replacement rate of RFA on the flexural strength.

As can be noticed from Fig 18, with the rise of LS content, the bending resistance of concrete was gradually increasing, and it arrived the peak value when the LS content is 20%, and then continued to increase the LS content, and the tensile strength also showed a downward trend. When the replacement rate of RFA was 0%, the flexural strength of concrete with 40% LS was 10.2% lower than that without LS. It can be seen from Fig 18 that the flexural strength of RFA concrete decreased with the rise of the replacement rate of RFA. When the content of LS was 0%, the flexural strength of RFA concrete with 30% substitution ratio decreased by 12.8% compared with that without LS. According to Fig 18, a proper amount of LS could effectively improve the low strength of RFA and other defects, but an excessive amount of LS would be counterproductive, which was basically consistent with the splitting tensile strength results. The cause analysis was basically similar to the splitting tensile strength mechanism, which would not be elaborated here. This observation is in line with Rahman et al., who found that 20% LS maximized flexural performance due to optimized C-S-H gel formation [21]. The decreasing trend with higher RFA levels confirms the conclusions of Chen et al., who noted similar reductions due to poor gradation and weak bonding in RFA concrete [31].

#### 3.4.2. Flexural strength and compressive strength of cubes.

**Fig 19:**
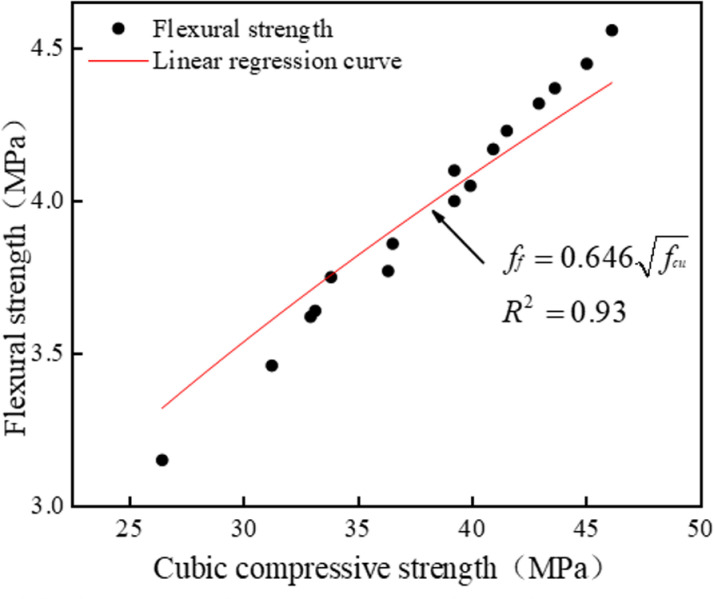
Relationship between flexural strength and cubic compressive strength.


The concrete flexural strength test is generally used to check the maximum load that the test block can withstand in the bending moment per unit area. A number of scholars have performed some studies on the relationship between the cubic compressive strength and flexural strength [48,49], and the flexural strength value of concrete is generally 1/5–1/10 of the cubic compressive strength value. The United States Concrete Institute (ACI) calculates the formula between the cubic compressive strength (*f*_*cu*_) and flexural strength (*f*_*f*_) as: $\begin{array}{c} ff=0.62\sqrt{f_{cu}} \end{array}$, European Concrete Board gets the relationship between the two for: $\begin{array}{c} ff=0.89\sqrt{f_{cu}} \end{array}$. With the help of the empirical formulas proposed by the above two countries, hypothesis formula $\begin{array}{c} ff=A\sqrt{f_{cu}} \end{array}$, linear regression analysis was performed on the flexural strength and cubic compressive strength of LSRFAC, as seen in Fig 19. The relationship between *f*_*f*_ and *f*_*cu*_ is obtained as shown in Eq. (2):

$$\begin{array}{c} ff=0.646\sqrt{f_{cu}} \end{array}$$ (2)

**Fig 20:**
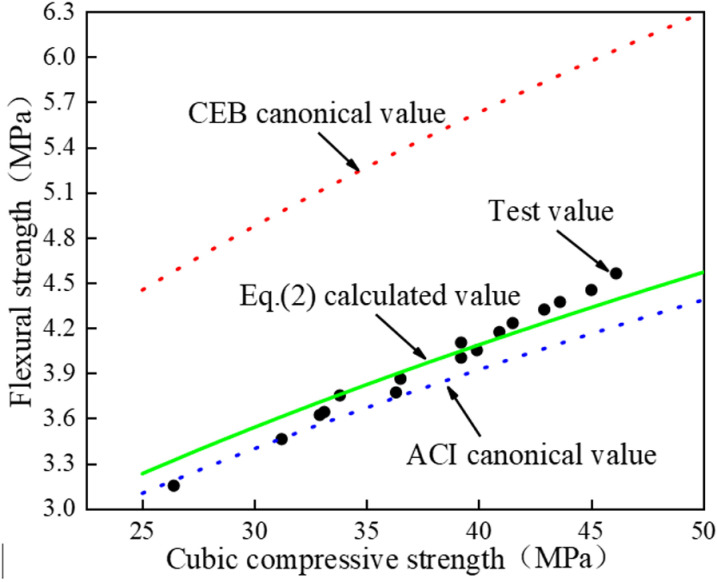
Comparison of flexural strength test values, standard values and calculated values.

The flexural strength test values, ACI and CEB standard values, and the calculated values of Eq. (2) were compared, as illustrated in Fig 20, the calculated values of Eq. (2) are very close to the experimental values, which also indicates that the linear regression works well. At the same time, the calculated value of Eq. (2) is located between the calculated value of ACI and CEB formula, which is close to the formula curve proposed by ACI, and is quite different from the formula curve proposed by CEB, which also indirectly indicates that the formula proposed by CEB is not suitable for calculating the flexural strength value of this test.

The findings of this study have important implications for sustainable construction. The use of LS to replace cement not only helps to reduce the accumulation of industrial waste, but also effectively reduces carbon emissions during cement production and promotes the development of green buildings. Meanwhile, the use of recycled aggregates instead of natural river sand can reduce the accumulation of construction waste and contribute to the recycling of resources. These results provide new ideas and practical basis for future sustainable building projects.

Based on the results of this study, future research can further focus on the assessment of LSRFAC’s durability in complex environments, reveal its enhancement mechanism by combining microstructural analysis, and explore the synergistic optimization effect of lithium slag with other mineral admixtures or nanomaterials. At the same time, we can verify the engineering applicability of LSRFAC through on-site application tests, carry out life cycle assessment to quantify its environmental benefits, and study ways to improve the quality of recycled fine aggregates, so as to comprehensively promote the application and development of LSRFAC in the field of green building materials.

It should be noted that the flexural strength prediction formula derived in this study is based solely on test results from lithium slag recycled fine aggregate concrete (LSRFAC). Due to the unique interactions between LS and RFA, the applicability of this formula to other concrete mixtures containing different supplementary cementitious materials or aggregate types remains uncertain. Further experimental studies are necessary to verify its generalizability.

## 4. Conclusions

This present study systematically surveyed the influences of replacing cement cementitious materials with different proportions of LS and replacing RS with different proportions of RFA on the mechanical properties of cementitious composites, and the main findings are as follows:

[1] The optimal mechanical performance was achieved when LS replaced 20% of the cement and RFA replaced 20% of river sand. Under this mix ratio, the compressive strength increased by 4.08% compared to the reference concrete, demonstrating that moderate LS content can effectively offset the negative impact of RFA on strength.
[2] While LS had minimal effect on slump at ≤20%, higher LS and RFA contents significantly reduced workability. A maximum slump reduction of 44.45% was observed when both LS and RFA were used at high levels.
[3] The use of LS and RFA promotes sustainable construction by reducing cement and natural sand consumption, lowering CO₂ emissions, and repurposing industrial and construction waste. The developed concrete is particularly suitable for non-structural and secondary structural applications, such as pavements, sidewalks, rural housing, and eco-friendly blocks
[4] This study focused primarily on early-age mechanical properties. Durability aspects—such as freeze-thaw resistance, shrinkage, permeability, and long-term performance—were not evaluated and should be considered in future investigations.
[5] Further research should include long-term durability tests, field-scale trials, and life-cycle assessments to confirm the real-world applicability of LSRFAC and deepen understanding of its performance in varied environmental conditions.
